# A Latent Profile Analysis of Beliefs about Alzheimer's Disease Risk Factors and Prevention Strategies among Americans Aged 50+

**DOI:** 10.1002/alz70860_105598

**Published:** 2025-12-23

**Authors:** Elyse Couch, Fangli Geng, Eric Jutkowitz, Emmanuelle Belanger

**Affiliations:** ^1^ Brown University, Providence, RI, USA

## Abstract

**Background:**

This study aimed to identify distinct patterns of beliefs regarding the importance of Alzheimer's disease (AD) risk factors and the effectiveness of prevention strategies among Americans aged 50+. This information could be used to develop public health interventions to increase engagement in dementia prevention strategies.

**Method:**

We analyzed data from 1,819 adults who participated in a single 2010 Health and Retirement Study experimental module on AD knowledge and beliefs. On a 3‐point Likert scale, participants rated the importance of AD risk factors (stress and genetics) for causing AD (1 = not important; 3 = very important) and the effectiveness of prevention strategies (physical activity, mental activity, diet, vitamins) (1 = not effective; 3 = very effective). We performed an unweighted latent profile analysis to identify distinct profiles of beliefs about AD risk factors and prevention strategies and multinomial regression to identify demographic predictors of profile membership.

**Results:**

We identified three distinct belief profiles. Profile 1 (63% of participants) were uncertain about the importance of risk factors and the effectiveness of prevention strategies; Profile 2 (15% of participants) strongly believed in the effectiveness of all prevention strategies, and Profile 3 (21% of participants) rated genetics as the most important risk factor but did not believe in the effectiveness of prevention strategies. Having a master's degree or above was associated with Profile 1 membership. Black participants were more likely than White participants to belong to Profiles 2 or 3 (RRR: 2.23 and 2.36, *p* <0.001). However, personal experience with AD was not associated with Profile membership. Compared with Profile 1, Profile 3 members had lower odds of wanting to learn their future AD risk (OR: 0.72, *p* = 0.029), and Profile 2 members had higher odds of believing they would develop AD in the future (OR: 1.26, *p* = 0.05).

**Conclusions:**

We found three distinct profiles of beliefs about AD risk factors and prevention strategies. Demographic characteristics, such as education and race, were associated with profile membership but not experience with AD. Public health messaging about AD prevention should be tailored to the beliefs of different demographic groups.

